# Multivariate Exploratory Analysis of the Bulgarian Soil Quality Monitoring Network

**DOI:** 10.3390/molecules28166091

**Published:** 2023-08-16

**Authors:** Galina Yotova, Mariana Hristova, Monika Padareva, Vasil Simeonov, Nikolai Dinev, Stefan Tsakovski

**Affiliations:** 1 Faculty of Chemistry and Pharmacy, Sofia University “St. Kliment Ohridski”, 1 J. Bourchier Blvd., 1164 Sofia, Bulgaria; g.yotova@chem.uni-sofia.bg (G.Y.); m.padareva@abv.bg (M.P.); vsimeonov@chem.uni-sofia.bg (V.S.); 2 Institute of Soil Science, Agrotechnologies and Plant Protection “N. Poushkarov”, Agricultural Academy, 7 Bansko shose Str., 1331 Sofia, Bulgaria; marihristova@hotmail.com (M.H.); ndinev@itp.bg (N.D.)

**Keywords:** soil analysis, nutrients, potentially toxic elements, multivariate statistics, principal components analysis, cluster analysis, kriging, soil management

## Abstract

The goal of the present study is to assess the soil quality in Bulgaria using (i) an appropriate set of soil quality indicators, namely primary nutrients (C, N, P), acidity (pH), physical clay content and potentially toxic elements (PTEs: Cu, Zn, Cd, Pb, Ni, Cr, As, Hg) and (ii) respective data mining and modeling using chemometrical and geostatistical methods. It has been shown that five latent factors are responsible for the explanation of nearly 70% of the total variance of the data set available (principal components analysis) and each factor is identified in terms of its contribution to the formation of the overall soil quality—the mountain soil factor, the geogenic factor, the ore deposit factor, the low nutrition factor, and the mercury-specific factor. The obtained soil quality patterns were additionally confirmed via hierarchical cluster analysis. The spatial distribution of the patterns throughout the whole Bulgarian territory was visualized via the mapping of the factor scores for all identified latent factors. The mapping of identified soil quality patterns was used to outline regions where additional measures for the monitoring of the phytoavailability of PTEs were required. The suggested regions are located near to thermoelectric power plants and mining and metal production facilities and are characterized by intensive agricultural activity.

## 1. Introduction

Soil quality can be defined as the ability of the soil to perform functions for its intended use. The capacity of the soil to function involves the balance and integration of sustained biological productivity, environmental quality and plant and animal health [1]. Soil quality cannot be measured directly because of its broad and integrative factors related to different soil uses. Soil quality evaluations are based on soil indicator (attributes) measurements, which reflect the inherent soil properties. Human management and natural disturbances can lead to significant changes in soil properties, which require a soil monitoring network, including the proper selection of an indicator data set related to reliable soil quality assessment.

Some reviews of national soil monitoring networks in Europe reveal serious deficiencies in monitoring the functional capacity of different soils and their changes over time [2,3]. The inspection of national monitoring networks emphasizes clearly unbalanced data sets with a dominance of chemical parameters in soil at the expense of biological and physical indicators [3]. Some of the indicators related to a decline in soil biodiversity and soil erosion are measured very rarely, whereas those related to soil compaction and the decline in soil organic matter and soil contamination are measured at almost all sites [2]. The other important drawback of soil monitoring in Member States of EU is the lack of harmonization of their national networks [4] regarding their design [2,3], soil sampling density [3] and method of analysis [5]. The overcoming of the above-mentioned obstacles and the expansion of the scope of monitored soil functions could be achieved via the establishment of an EU-wide monitoring network, which is the aim of the European-scale LUCAS soil survey (Land Use and Coverage Area Frame Survey) [2].

The Bulgarian soil quality monitoring network is no exception, and it contains well-documented records concerning basic chemical soil indicators and potentially toxic elements. The good sampling density provides the opportunity for the reliable monitoring of some of the basic soil functions like primary productivity, nutrient cycling and soil contamination [3]. Usually, such data sets are used for establishing the geochemical background and threshold of chemical elements [6], but incorporating basic soil chemical indicators in data modeling could provide important information for the phytoavailability of PTEs. The prolonged existence of PTEs in the ecosystem poses a potential risk to human health, caused by the consumption of contaminated plants or the direct inhalation of soil particles [7]. Despite the lower levels of industrial pollution in recent years, it is essential not to underestimate the concerns related to PTEs [8]. Although PTEs like Cu, Mn and Zn are essential micronutrients for plants, their high concentrations could cause toxic effects and serious risks for the food chain [7].

Along with the selection and assessment of an optimal set of soil indicators [3], such a complex and multivariate task as soil quality assessment requires adequate data treatment. The application of multivariate statistical methods, like cluster analysis (CA) and principal components analysis (PCA), can not only reveal hidden interactions between different soil indicators but also identify the factors affecting soil quality, including natural processes and anthropogenic pressures [9,10,11,12,13,14,15,16,17,18,19,20,21]. In most of the aforementioned studies, multivariate exploratory approaches are used in order to reveal and estimate the anthropogenic sources of potentially toxic elements.

A solid approach for visualizing the regional distribution of soil indicators or achieved latent factors is geostatistics, namely kriging interpolation using geographical information system (GIS) techniques. This method is often used to illustrate the spatial distribution of soil indicators [11,15,18,19,20,21,22,23,24,25,26] and, less frequently, to project the spatial distribution of the identified latent factors on a map [10,14,23,27].

The aim of the present study is to reveal the latent factors controlling the soil quality of Bulgarian territories based on the soil indicators collected during the Bulgarian soil quality monitoring program. The GIS-based mapping of soil quality pattern distributions was carried out in order to evaluate the impact assessment of PTEs in agricultural land areas.

## 2. Results

### 2.1. Basic Statistics

The 347 topsoil samples of the Bulgarian soil quality monitoring network were analyzed for thirteen soil indicators, namely the pH, the main nutrients (organic carbon—C; total nitrogen—N; total phosphorus—P), physical clay content and eight potentially toxic elements (Cu, Zn, Cd, Pb, Ni, Cr, As, Hg). Thus, the input data matrix consists of 347 rows (objects) and 13 columns (variables). The basic statistics of the input data are provided in Table 1.

### 2.2. Chemometric Data Interpretation

Multivariate statistical methods (CA and PCA) were applied to uncover the relationships between three groups of parameters: basic soil characteristics (pH and physical clay), nutrient elements (C, N and P) and eight potentially toxic elements (Cu, Zn, Cd, Pb, Ni, Cr, As, Hg). First, PCA (Varimax rotation normalized mode) was performed. The PCA results show that the first five latent factors (PCs) explain more than 65% of the total variance. The factor loadings of the selected PCs are presented in Table 2.

**Figure 1:**
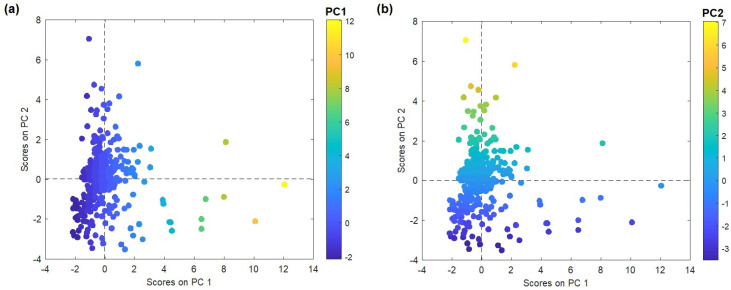
Plot of PC1 vs. PC2 factor scores: (**a**) colored by PC1 factor score values; (**b**) colored by PC2 factor score values.

The factor score plot presented in Figure 1 illustrates the distribution of sampling points according to the first two principal components. For sampling points with higher PC1 factor scores, the mountain soil pattern prevails (Figure 1a), while for points with higher PC2 factor scores, the geogenic origin dominates.

**Figure 2:**
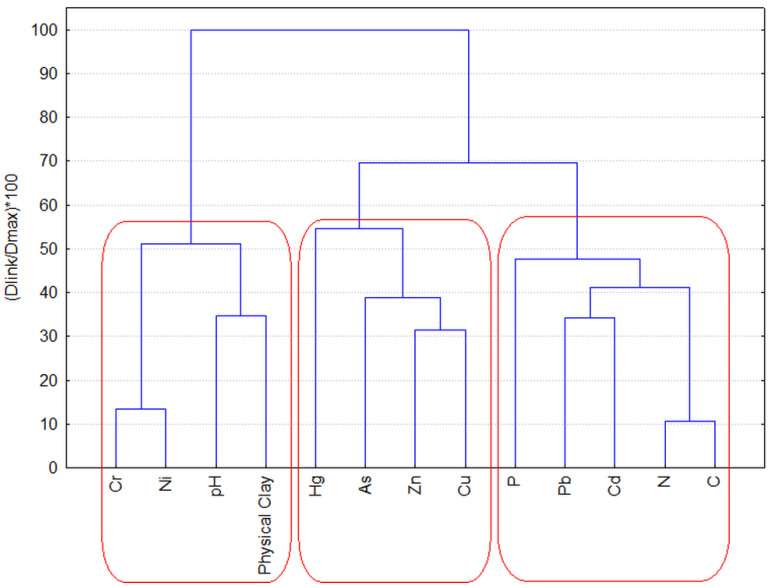
Hierarchical dendrogram of the clustering of the 13 soil indicators.

The relationships between soil parameters obtained in PCA were almost entirely confirmed via the application of hierarchical cluster analysis to the same data set (z-standardized input values, squared Euclidean distances as a measure of similarity and Ward’s method of linkage). The grouping of measured soil indicators is presented in Figure 2. Three major clusters are formed: C1 (C, N, Cd, Pb, P); C2 (Cu, Zn, As, Hg); and C3 (physical clay, pH, Ni, Cr).

### 2.3. Mapping of Principal Components

**Figure 3:**
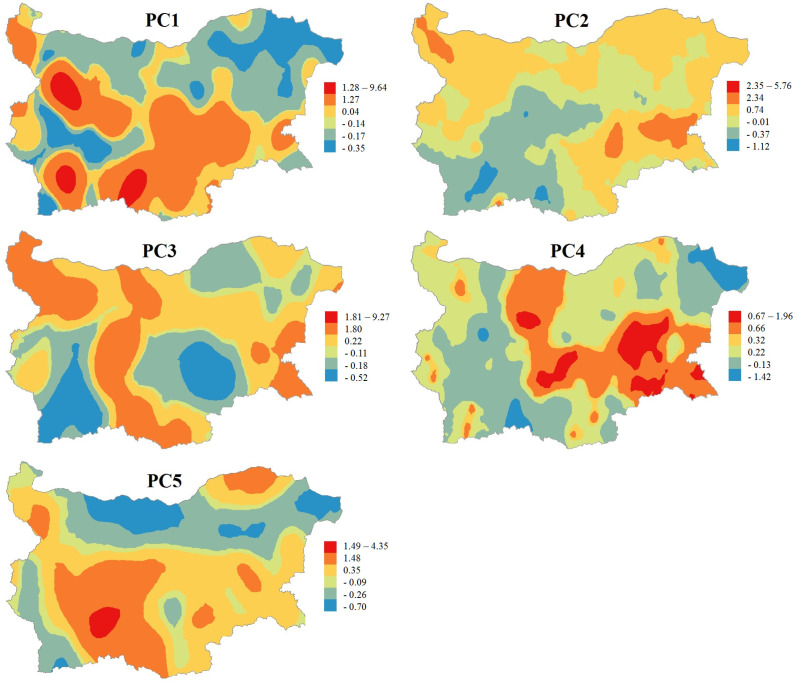
Spatial distribution of the principal components and soil sites determined using the PCA.

The next step of the study is to explore the spatial distribution of the principal components using GIS-based maps of the respective sampling sites’ factor scores. According to the factor scores of the 347 samples, kriging maps for each one of the five latent factors are presented (Figure 3). The spatial distribution of the principal components is an appropriate method for outlining the regions that are typical for a given factor and for clarifying the origin of a specific component.

## 3. Discussion

### 3.1. Basic Statistics

According to the mean values obtained from basic statistics, the soils studied have a medium content of C and N. The C/N ratio shows that most of the soils are characterized by high quality humus, type mul, in which stable organic humates predominate [28]. The confidence interval of the mean pH value characterizes the soils as neutral (from slightly acidic to slightly alkaline). The content of physical clay in most of the soil samples shows that they have good physical and physicomechanical properties, as only 7.8% of the samples were characterized as sands with a physical clay content of less than 20%. The concentration of potentially toxic elements in the samples from the Bulgarian soil quality monitoring network shows low levels of anthropogenic impact. Only 6.1% of the soil samples had PTE concentrations at higher values than the maximum allowable concentrations [29], and there were mainly located in the western and southeastern parts of Bulgaria. The observed exceedances (twenty-one in total) are as follows: five for Cu, one for Cd, three for Pb, three for Ni, three for Cr and six for As.

### 3.2. Chemometric Data Interpretation

The selected five latent factors (PCs) in the PCA analysis explain more than 65% of the total variance.

The first latent factor (PC1) explains 15.73% of the total variance of the data set. It reveals the relationship between the nutrient elements carbon and nitrogen and the metals Cd and Pb. Such a “pattern” can be related to mountain soils in the Bulgarian network, mainly belonging to the Balkan and Rhodope tectonic zones [30]. It is typical for such soils that the mobility of Cd and Pb is controlled by soil organic matter [21,31].

In PC2, significant loadings possess Ni, Cr and physical clay. This factor is conditionally named “geogenic” [23] and explains 15.37% of the data set variance. Many studies confirm that Ni and Cr content in soil depends on the soil formation and composition of the parent rock material [16,21]. The same conclusion is reported in soil quality studies where only metals are included as indicators [9,18,23,32]. It should be noted that the moderate presence of Cu, Zn and Hg in this factor is an indicator for a specific “natural” content of these metals, which originate in the Earth’s crust.

**Figure 4:**
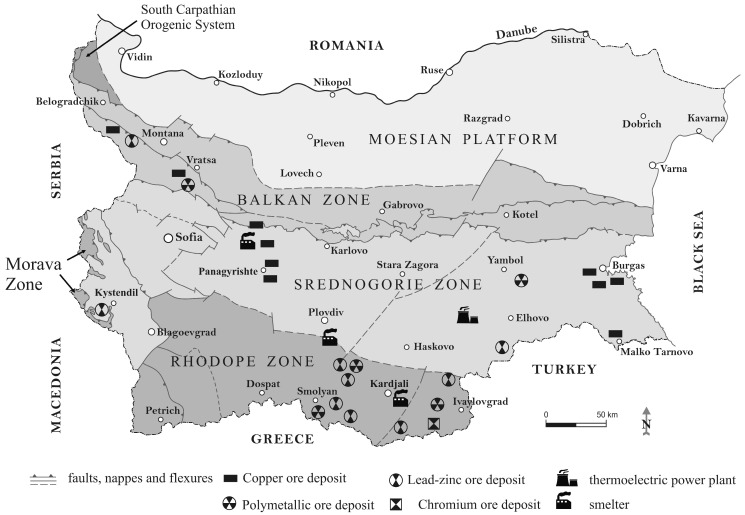
Simplified tectonic map of Bulgaria with major exploited ore deposits, metal smelters and thermoelectric power plant.

PC3 explains 12.21% of the total variance and can be conditionally named “ore deposits”. It resembles the relationship between the significantly contributing Cu, Zn and As and the moderately participating Pb. Usually, these elements are pollutants that originate mainly from industrial, mining and agricultural activity [19,20,21,32,33], but their significant presence in the Earth’s crust may be related to mineral ore deposits (Figure 4). Arsenic is generally recovered from sludge and flue dust in smelters. Furthermore, there are arsenic emissions in coal-burning areas. Its presence in non-ferrous ores is generally regarded to be an environmental problem and not a benefit [34].

The behavior of carbon and nitrogen is quite interesting. They participate significantly in the formation of two factors—PC1 and PC4. In the fourth factor, C and N are logically connected with P also being a typical nutrient (all three elements have negative factor loadings in PC4). This factor explains 11.74% of the data set variance and can conditionally be named “low nutrition”. The appearance of such a factor in the data structure modeling via PCA could be related to the low humus content in soils, as well as to soils subject to nutrient components exhaustion at intensive agriculture.

The fifth factor (10.25%) reveals a positive relationship between physical clay and pH, which contradicts previous studies [11,35]. The significant factor loadings in PC5 lead to the conclusion that in the Bulgarian soil quality network, high concentrations of Hg are observed in sandy acidic soils. Since anthropogenic sources of Hg are limited within the territory of Bulgaria, the elevated values of Hg can be considered baseline levels, especially in the soils mentioned above. This can be confirmed via the comparison of the mean values of Hg in the FOREGS geochemical database (0.016 mg kg^−1^) [36] and those in this data set (0.15 mg kg^−1^).

In general, the results of both multivariate statistical approaches reveal similar patterns of similarity between the soil indicators used—one can identify the same factors responsible for the linkage and correlation between soil indicators, namely geogenic (cluster 3), nutritional (cluster 1) and ore deposits (cluster 2). By using PCA the data interpretation could be additionally improved by commenting on some other features of the data structure, like the specification of the mountain soil type and the unexpected mercury content.

### 3.3. Mapping of Principal Components

For better description and interpretation of the obtained results a simplified tectonic map of Bulgaria [37] is presented on Figure 4. Moreover, the major exploited ore deposits, as well as the biggest metal smelters and thermoelectric power plant are also indicated.

The first latent factor is related to mountain soils pattern. As expected, sites with the highest factor scores of PC1 are situated mainly in the largest Bulgarian mountains—Rila, Pirin, the Rhodopes, and the Balkans. This spatial distribution confirms that the high content of Pb and Cd is prevalent in mountain soils, such as Cambisols and Luvisols, and is not due to industrial activity in these regions. Moreover, the highest factor scores of PC1 are observed in areas with lead–zinc ore deposits (the central Rhodope zone and the western part of the Balkan zone) and copper mining areas (western part of the Balkan zone and the eastern part of the Srednogorie zone). It should be mentioned that the high factor scores in the central Srednogorie zone may be due to anthropogenic impact caused by contamination with Cd from the biggest coal-fired power plant in Bulgaria (Maritsa Iztok complex) (Figure 4) [38].

The geogenic factor (PC2) has higher factor scores in soils from the South Carpathian orogenic system, western Balkan Zone and eastern part of Srednogorie Zone. Most of the samples are from Chernozems and Vertisols soil types, which are characterized with high physical clay content. The sample sites with low factor scores of PC2 are mainly of Fluvisol, Cambisol and Leptosol soil types with high sand content. The abovementioned soil patterns correspond well with the origin and mapping of the “geogenic” principal component. In previous studies [38], it has been discussed that the presence of ophiolite and diabase–phylitoide complexes in the western Balkan Zone and igneous basic rocks in the western and eastern part of the Srednogorie Zone [39] confirmed the parent rock control of Ni and Cr. Again, high factor scores can be observed in the central Srednogorie zone that could be due to contamination caused by the Maritsa Iztok complex (Figure 4).

The PC3 map presents a spatial distribution of elevated factor scores regions coinciding with the ore deposits in Bulgaria, mainly copper, lead–zinc and polymetallic ores (Figure 4). Dimitrova et al. [40] reported higher concentrations of Cu, Zn, As and Pb in northwestern Bulgarian soils caused by mining activities in the region. It has already been mentioned that the largest mining areas are in the central Rhodope zone, the western part of the Balkan zone and the eastern part of the Srednogorie zone, but unexploited ore deposits are also located in the southern central part of the Moesian platform [41].

Most of the soil samples with the lowest factor scores of PC4, which indicates high nutrient content, are from the most fertile and agricultural (arable) type—Chernozem. These sites are the best for agricultural use, such as Dobrudzha (northeastern Bulgaria) and the valleys of the rivers Iskar, Struma and Vacha. The average concentration of nutrients for the samples with the lowest factor scores (below −1) comprises a very high content of C and P and a high content of N, whereas those with the highest factor scores (above 1) have an average content of C and low content of N and P.

The soil samples, which have high scores in the Hg-specific factor (PC5) are mainly derivedfrom three soil types: Cambisols, Leptosols and Fluvisols. The parent material of the first two types are non-carbonate rocks, which have high acidity and low physical clay content (a high content of sand). The mean mercury concentration in the Bulgarian soil quality monitoring network (0.15 mg kg^−1^) is quite high and follows the trend reported in other European studies, where soil mercury content increases from northern to southern Europe [42]. The high factor scores in the western Balkan zone and the central Rhodope zone are in a good agreement with the ore deposits, as the elevated Hg soil content in central Srednogorie region is presumably also due to the coal-fired power plant [38]. The northeastern part of Moesian platform (near the town of Silistra) is not an anthropogenically influenced area, which is an indication that such high Hg levels could be considered as baseline ones for the respective region.

The simultaneous consideration of the spatial distribution of soil quality patterns and arable land coverage could outline the regions where additional measures for the monitoring of the phytoavailability of PTEs and their transfer to plants are appropriate. The elevated concentrations of Cd and Pb (associated with PC1) are mainly found in mountain regions with limited agricultural activity. Concerning geogenic PTEs, in PC2, Ni and Cr require special measures for the monitoring of their phytoavailability only in the central Srednogorie region, which is affected by the Maritsa Iztok thermoelectric power plant. The most anthropogenically influenced soil quality pattern (PC3) presenting ore deposits and related mining and metal production activities has to be examined carefully. Excluding the regions with elevated factor scores of geogenic factor (PC2), which could be an indication for the higher regional background concentrations of Cu, Zn, As and Pb, the western Srednogorie zone remains an anthropogenically polluted region with significant agricultural activities. The previous study in this region [7] outlines EDTA soil extraction as a reliable procedure for the phytoavailability estimation of As, Cd, Cr, Cu, Mn and Pb. The soil–plant transfer of the main contaminants of Cu and As around the copper mining and smelter factories is controlled mainly by soil pH, total organic matter and CaCO_3_. The transfer coefficient of Cu is positively correlated with soil pH and CaCO_3_ and negatively with total organic matter. These relationships can be explained by the increasing stability of the Cu(II)-EDTA complex at higher pH values and the binding ability of organic soil fractions. The reason for positive correlation between soil–plant transfer and total organic matter can be found in the dissolution of As, which is bound to soil humic substances at the pH of the EDTA leaching procedure. The soil–plant transfers of Zn and Pb are controlled by Al and Fe soil contents, respectively. The only region with a significant presence of Hg-specific soil quality patterns (PC5) and with significant agricultural activity is the aforementioned central Srednogorie region, which is affected by the Maritsa Iztok thermoelectric power plant. It can be concluded that the soil management of the western and central Srednogorie zone needs additional measures for the monitoring of PTEs’ phytoavailability.

## 4. Materials and Methods

### 4.1. Sampling and Chemical Analysis

**Figure 5:**
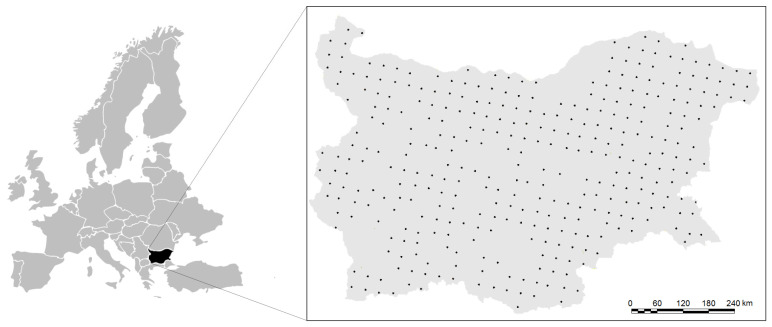
Locations of sampling points of the Bulgarian soil quality monitoring network.

In general, the sampling and sample preparation procedures are fully described in national directives and documents, according to international standards (ISO 10381-2:2005, ISO 10381-4:2005 and ISO 11464:2006) [43,44,45]. The 347 topsoil samples of the Bulgarian soil quality monitoring network were collected at depths of 0–20 cm and in the intersections of an orthogonal 16 km grid across the whole country (Figure 5).

Soil sampling was performed in the 2004–2009 period, and the soil samples were taken from different types of land with different usages. For each sample, a total of 13 soil indicators were measured as follows: pH, main nutrients (organic carbon—C; total nitrogen—N; total phosphorus—P), physical clay content and 8 potentially toxic elements (Cu, Zn, Cd, Pb, Ni, Cr, As, Hg).

The main soil indicators (pH, physical clay) and the nutrients were determined via the following analytical methods: pH (ISO 10390:2005) [46]; organic carbon via sulfochoromic oxidation (ISO 14235:2002) [47]; total nitrogen via the modified Kjeldahl method (ISO 11261:2002) [48]; and total phosphorus via a validated method based on acid mixture microwave digestion and analyzed through ICP-OES. The particle size distribution was determined through the method of sieving and sedimentation (ISO 11277:2009) [49]. Only a fraction of the physical clay (percentage of the soil particles with diameters of less than 0.02 mm) was used for statistical modeling.

The soil aqua regia extraction of potentially toxic elements was performed according to ISO 11466:1995 [50]. Six of the elements (Cu, Zn, Cd, Pb, Ni, and Cr) were determined via atomic absorption spectrometry (ISO 11047:1998) [51], and As and Hg were determined using ICP-MS, according to the CEN/TS 16171:2012 [52] standard.

### 4.2. Data Analysis Methods

For the chemometric assessment and interpretation of the obtained data, two multivariate statistical approaches were used: cluster analysis [53] and principal components analysis [54].

Cluster analysis (CA) is a well-known and widely used classification approach in chemometric and environmetric studies, with its hierarchical and non-hierarchical algorithms. The main goal of the hierarchical agglomerative cluster analysis was to spontaneously classify the data into groups of similarity (clusters). Usually, the sampling sites in traditional monitoring surveys are considered objects for classification, but it is also possible to search for links between the variables (different soil quality indicators) that characterize them. A preliminary step of CA is the normalization of the raw input data (e.g., autoscaling or z-transformation) in order to avoid the influence of the different range of chemical dimensions (concentration). As a result, normalized dimensionless numbers replaced the real data values. Then, the distance between the objects (or the variables) of classification was determined, usually by applying squared Euclidean distances as a similarity measure. There are a wide variety of hierarchical algorithms for object linkage—the single linkage, the complete linkage or the average linkage methods—but Ward’s method is predominantly used, because it achieved balanced clustering, while taking into account the intra- and inter-cluster distances. The CA results are normally depicted using a tree-like scheme with a hierarchical structure called a dendrogram.

Principal components analysis (PCA) is widely used as a dimension-reducing, -modeling and -display method, which allows the estimation of internal relations in the data set. PCA enables the reduction of the coordinate system of the variables in the direction of the highest variance. The input variables are converted into new ones, which are better descriptors of the data structure. The new variables, called principal components (PCs) or latent factors, are a linear combination of the original variables. Usually, just a few of the latent factors account for a large part of the data set variation. Thus, the data structure in a reduced variable space can be observed and interpreted. As a result of PCA, the autoscaled original data matrix (**D**) was transformed into a product of two matrices as follows:

$$D=UV^{T}+E$$

where **V^T^** is factor loadings matrix, revealing the contribution of each one of the original variables (indicators) to the newly formed principal components; **U** is factor score matrix, providing the new coordinates of each object (sampling point) in the new space of principal components; and **E** is residual matrix.

In this study, the Varimax rotation mode of PCA was applied for the better interpretation of the system (soil quality), as it increases the role of the soil indicators with higher impacts on the formation of principal components and decreases the role of soil indicators with lower impacts.

A GIS-based approach was chosen to present the spatial distribution of principal components achieved via PCA analysis. Principal components maps were plotted using the kriging interpolation of principal component factor scores (row of matrix **U**) of all sampling points from the Bulgarian soil quality monitoring network.

## 5. Conclusions

The present study is a pioneer effort to integrate all aspects of a proper soil quality assessment for the territory of Bulgaria. The use of state-of-the-art monitoring and analytical and chemometric approaches made it possible to better understand and classify the factors related to different soil patterns in the country, such as spatial, geogenic, nutritional and anthropogenic. The mapping, based on the chemometric results, offers an additional insight into the soil specificity throughout the country through the specific distribution of the identified soil quality patterns. The content of potentially toxic elements in the soil samples reveals the low level of anthropogenic impact, as higher values of Ni and Cr are of geogenic origin and higher values of Pb and Cd are mainly observed in mountain regions; higher values of Cu, Zn and As are mainly found in regions with ore deposits; and higher values Hg can mainly be located in site-specific sandy acidic soils.

Such an assessment of the soil quality of a whole country requires many different prerequisites, namely a well-organized soil sampling network; well-trained analytical staff able to perform advanced determinations of all specific soil quality indicators—both structural and chemical; and the correct classification, modeling, and interpretation of the collected data. The proposed methodology is able to outline the anthropogenically influenced regions characterized by intensive agricultural activity where additional measures for the impact assessment of PTEs are required.

The results obtained via data mining and modeling ensure better soil quality management and sustainability as well as efficient agricultural decision making.

## Figures and Tables

**Table 1:** Basic statistics of the input data set (*n* = 347).

	Dimension	Mean	St. Dev.	Median	Minimum	Maximum
C	g kg^−1^	18.8	10.7	16.0	0.31	113
N	g kg^−1^	1.81	0.96	1.60	0.40	9.91
P	mg kg^−1^	881	585	740	199	4634
Physical clay	%	51.6	19.6	57.5	9.79	83.0
pH	-	6.78	0.98	6.80	3.80	8.80
Cu	mg kg^−1^	31.4	30.8	23.7	3.60	351
Zn	mg kg^−1^	63.5	22.3	64.3	1.26	162
Cd	mg kg^−1^	0.23	0.31	0.16	0.02	4.32
Pb	mg kg^−1^	20.5	17.8	16.8	3.07	200
Ni	mg kg^−1^	35.8	20.2	35.4	1.20	208
Cr	mg kg^−1^	53.0	34.7	45.4	2.30	213
As	mg kg^−1^	8.21	11.3	6.70	0.04	159
Hg	mg kg^−1^	0.15	0.13	0.12	0.01	0.97

**Table 2:** Factor loadings for five latent factors and their conditional names.

	PC1	PC2	PC3	PC4	PC5
C	0.63 ^a^	−0.04	0.00	−0.59	0.20
N	**0.73** ^b^	0.00	0.09	−0.52	0.00
P	−0.02	0.00	0.08	**−0.83**	−0.08
Physical Clay	−0.02	0.50	0.05	0.06	−0.39
pH	0.10	0.24	0.04	0.16	**−0.79**
Cu	−0.05	0.24	**0.72**	0.06	0.08
Zn	0.16	0.14	0.70	−0.28	−0.04
Cd	**0.78**	−0.01	−0.03	0.15	−0.08
Pb	0.64	−0.09	0.26	−0.01	0.07
Ni	−0.05	**0.90**	0.05	−0.03	−0.11
Cr	−0.04	**0.87**	0.10	0.00	0.13
As	0.13	−0.12	0.70	0.02	−0.07
Hg	0.15	0.18	0.03	0.30	0.68
Expl.Var.%	15.73	15.37	12.21	11.74	10.25
Conditional name	Mountain soil	Geogenic	Ore deposits	Low nutrition	Hg-specific

^a^ Factor loadings with absolute values between 0.4–0.7 are underlined. ^b^ Factor loadings with absolute values above 0.7 are shown in bold.

## Data Availability

The data presented in this study are available on request from the authors.

## Sample Availability

Samples of the compounds are not available.
